# Cognitive Therapy vs. Short‐Term Dynamic Psychotherapy in a Community Mental Health Setting: A Preliminary Analysis of Effects in Several Severity Domains

**DOI:** 10.1002/cpp.70254

**Published:** 2026-03-15

**Authors:** Mary Beth Connolly Gibbons, Nic Kodkany, Yaz Liow, Paul Crits‐Christoph

**Affiliations:** ^1^ Department of Psychiatry University of Pennsylvania Perelman School of Medicine Philadelphia Pennsylvania USA

**Keywords:** cognitive therapy, dynamic therapy, functioning, noninferiority, symptom severity

## Abstract

**Objective:**

To investigate the effectiveness of cognitive therapy (CT) and short‐term dynamic psychotherapy (STDP) among patients with greater severity, defined in several ways, in a community mental health setting.

**Method:**

Using data from a randomized noninferiority trial, we examined five baseline severity variables as potential moderators of treatment effects: depressive symptoms, interpersonal problems, mental functioning, physical functioning and quality of life. The outcome was a change in depressive symptoms over the course of up to 5 months of treatment. Linear mixed‐effects models were implemented to compare slopes of change over time. We hypothesized that CT would be superior to STDP for those with severe depressive symptoms, severe mental and physical functioning and severely impaired quality of life, but STDP would be superior to CT for those with severe interpersonal problems.

**Results:**

There was no evidence of superiority within any of the five severity domains.

**Conclusions:**

Findings inform the comparative effectiveness of CT and STDP among patients with greater severity and extend prior work by incorporating interpersonal, functioning and quality of life severity indicators.

## Summary

- There is no evidence that patients with more severe depressive symptoms will benefit more from cognitive therapy compared with short‐term dynamic psychotherapy.
- Psychotherapists should expect both cognitive therapy and short‐term dynamic psychotherapy to be equally effective for the treatment of patients with more severe mental and physical functioning.
- Patients with poor quality of life benefit equally from cognitive and dynamic psychotherapies.
- There is no evidence that patients with more severe interpersonal problems benefit more from short‐term dynamic psychotherapy compared with cognitive therapy.

In comparison with relatively mild symptoms, the presence of moderate to severe depressive symptoms among individuals with major depressive disorder (MDD) is associated with a variety of negative outcomes. Specifically, a meta‐analysis of suicidality prevalence in MDD reported that severe depressive/psychotic features predicted suicidal ideation and prospective risk of suicide (Cai et al. 2021). Further, Ding et al. (2024) recently demonstrated that greater baseline depressive severity predicted persistence of suicidal ideation trajectories among those with MDD. Higher levels of depressive symptoms among those with MDD have also been found to be associated with overall work impairment and relatively poor cognitive functioning (Sumiyoshi et al. 2019). Increased symptom severity of MDD is additionally associated with greater health care utilization and higher medical and indirect costs (Culpepper et al. 2024).

The notion of ‘severity’, however, should not be restricted to symptom burden alone. Interpersonal dysfunction, poor functioning and decreased quality of life represent complementary severity indicators that are associated with adverse outcomes. A recent meta‐analysis aggregated effect sizes across 38 studies and found an average 0.41 correlation between interpersonal distress and problems and severity of noninterpersonal mental health measures (e.g., self‐esteem) among individuals with depressive disorders (Gómez Penedo, Areas, and Flückiger 2025). Further, another meta‐analysis found that more severe interpersonal distress was associated with relatively poor treatment outcomes (Gómez Penedo, Meglio, et al. 2025). In addition, a high level of interpersonal problems is an identified risk factor for suicide attempts, particularly for women (Miranda‐Mendizabal et al. 2019).

Functioning and quality of life can also be considered significant indices of severity, and they are distinct in terms of how they are operationalized. Assessment of functioning is typically designed to ask questions about limitations that may occur in various physical and social activities. Quality of life measures, in contrast, focus on the degree of satisfaction in different areas of one's life. Although functioning and quality of life typically improve with treatment, these dimensions often remain impaired relative to healthy controls. Such impairments persist over time, especially if remission of symptoms has not been achieved (Ishak et al. 2015). These findings suggest that it is clinically important to understand the relative effectiveness of different treatment modalities for those with MDD who not only have severe depressive symptoms but also severe interpersonal problems and severe impairments in functioning and quality of life.

Multiple psychotherapy modalities have demonstrated efficacy in the treatment of adult depression (Cuijpers et al. 2021). Among those with moderate to severe depressive symptoms, cognitive therapy (CT) has shown efficacy (DeRubeis et al. 2005; Hollon et al. 2005). Though comparative trials and meta‐analyses indicate that short‐term psychodynamic therapy (STDP) can be effective for depression and equivalent to other psychotherapies (Driessen et al. 2010; Driessen et al. 2013; Caselli et al. 2023; Connolly Gibbons et al. 2016; Steinert et al. 2017), there are few rigorous head‐to‐head tests in severe presentations. An individual patient meta‐analysis comprised of 10 studies failed to find that treatment type moderated the association of baseline severity of interpersonal distress with change in depressive symptoms from baseline to post‐treatment, 12‐month follow‐up or 24‐month follow‐up (Gómez Penedo, Meglio, et al. 2025). Only two of the 10 studies, however, compared a dynamically oriented treatment to a cognitive‐behavioural treatment (CBT). Both studies were conducted in the United Kingdom. Barkham, Rees, et al. (1996) compared a psychodynamic‐interpersonal therapy to CBT. The sample size of this study was very small: 36 patients were randomized to two variations (8 and 16 sessions) of the two treatment types. The second study focused on moderate to severe depression and randomized patients to low‐intensity therapy, dynamic‐interpersonal therapy or CBT (Fonagy et al. 2020). The sample size of the CBT group was small (*n =* 20). In addition, the mean baseline scores on the 17‐item Hamilton Rating Scale for Depression (HAM‐D‐17; Hamilton 1960) were indicative of moderate severity (between 18 and 19). Thus, the evidence base for understanding the relative effectiveness of CT and STDP for severe MDD, regardless of the severity indicator, is sparse. In fact, no studies have compared a psychodynamic therapy to CT for those with only severe depressive symptoms. Specifically, no studies have focused on Luborsky's (1984) supportive‐expressive version of dynamic therapy for more severely depressed patients. In addition, no studies have examined the effectiveness of STDP or CT for severely depressed patients in the context of a US community mental health clinic (CMHC) or have compared these treatments for those who have severe impairments in interpersonal problems, functioning and quality of life.

The theoretical models that guide STDP would suggest that this treatment may be especially effective for MDD patients who have severe interpersonal problems. Severity of interpersonal problems has been found to be associated with a history of trauma within the context of patients with depression presenting for treatment at a community mental health centre (Goldstein et al. 2023). Psychodynamic theorists and clinicians have long focused on the understanding and treatment of trauma, with different psychodynamic approaches placing more emphasis on internal defences, interpersonal interactions or developmental factors as a lens to explore the impact of trauma. Regardless of the specific dynamic approach, addressing interpersonal themes in dynamic therapy sessions has been associated with improved alliance and outcomes (Crits‐Christoph et al. 1988; Crits‐Christoph et al. 1993; Norville et al. 1996; Piper et al. 1993; Silberschatz 2017). Presumably, patients with more severe interpersonal problems would be more likely to have such themes addressed in dynamic therapy. When severe interpersonal problems arise with the therapist (i.e., transference); however, it appears to be contraindicated to address such themes directly with transference interpretations (Connolly et al. 1999; Høglend et al. 2006). The implication of these studies is that addressing interpersonal problems can be effective. However, when more severe interpersonal problems are acted out in the therapeutic relationship, dynamic therapists must skilfully apply supportive techniques and focus on themes with significant people in the patient's world, rather than transference interpretations. If implemented skilfully, therefore, STDP may be particularly effective for MDD patients with severe interpersonal problems. Improvement in interpersonal problems has been linked to improvement in depressive symptoms. Therefore, addressing interpersonal problems may not only improve such problems but also carry over to the relief of depressive symptoms (Høstmælingen et al. 2025).

Regarding patients with severe functional and quality of life impairments in MDD, there are a couple of reasons why CT may be particularly helpful. For one, the behavioural activation strategies used during the early phase of CT target the type of functional impairments that are a major element of quality of life (e.g., limited activities, limited physical exercise and decreased interactions with family, friends, etc.). Improvements in these activities may also lead to a more positive quality of life. A second reason is that research has indicated that greater impairment in quality of life in those with MDD is associated with the use of negative coping strategies (Holubova et al. 2017). CT focuses on building more positive coping strategies that in turn may improve functioning and quality of life among patients with MDD who have severe impairments.

The present analyses build on a randomized noninferiority trial conducted in a community mental health setting directly comparing CT and STDP for MDD (Connolly Gibbons et al. 2016). The primary finding of the study was that STDP was noninferior to CT on primary (depressive symptoms) and secondary (functioning, quality of life) outcome measures. Using this community clinic randomized trial dataset, we examined potential severity moderators of the effectiveness of CT and STDP. Severity was defined in five ways: higher depressive symptoms, greater interpersonal problems, poor mental functioning, poor physical functioning and poor quality of life. Based on prior work demonstrating robust CT effects in moderate‐to‐severe depression (defined as a HAM‐D‐17 of 20 or greater; DeRubeis et al. 2005; Hollon et al. 2005), we tested the hypothesis that CT would produce faster improvement in depressive symptoms over time than STDP for patients who enter treatment with more severe depressive symptoms. We also hypothesized that CT would have superior outcomes compared with STDP for patients with more severe mental functioning, physical functioning and quality of life impairments. For those with severe interpersonal problems, we hypothesized that STDP would show greater changes in depressive symptoms than CT.

## 1 Methods

All study procedures were conducted in compliance with the University of Pennsylvania Institutional Review Board and written informed consent was obtained from all patients. The overarching clinical trial can be found at ClinicalTrials.gov (NCT01207271). Further details of the methods are outlined by Connolly Gibbons et al. (2014).

### 1.1 Setting

We collected all data in conjunction with NHS Human Services (NHS), a large, non‐profit, community‐based organization that provides mental health and substance abuse services. NHS works primarily with patients on Medicaid/Medicare in the mid‐Atlantic region of the United States. The trial was conducted at an outpatient CMHC in Sharon Hill, Pennsylvania. The clinic employs 80 therapists and three to four psychiatrists and serves about 4900 patients per year.

### 1.2 Participants

#### 1.2.1 Patients

Individuals seeking services for depression at the CMHC were recruited for the randomized noninferiority trial. All adult patients who attended an intake assessment at the clinic completed the Quick Inventory for Depressive Symptomatology (QIDS; Rush et al. 2003). The intake clinician completed a short eligibility checklist and scored the QIDS. Patients were referred to the research team if they were able to read English at the fourth‐grade level, were between the ages of 18 and 65 and scored 11 or higher on the QIDS. For eligible patients, a member of the research team conducted a brief phone screening and scheduled a baseline assessment at the CMHC.

At the baseline assessment, a research assistant collected informed consent and self‐report questionnaires. A blinded clinical evaluator conducted the Hamilton Depression Rating Scale (HAM‐D; Hamilton 1960) and the Structured Clinical Interview for the DSM‐IV Axis I disorders interview (SCID; First et al. 1997). Patients were excluded if they had (a) a diagnosis of schizophrenia; (b) a diagnosis of psychosis or MDD with psychotic features; (c) a seizure disorder; (d) a diagnosis of bipolar disorder; (e) a diagnosis of depression due to organic pathology; (f) symptoms of substance or alcohol use disorder that would necessitate immediate referral to substance abuse treatment; (g) suicidal ideation judged by the clinic to require psychotherapy more frequent than once a week; and/or (h) problems requiring immediate referral to a partial or inpatient hospitalization program. Patients were allowed psychotropic medications during the trial, as use of these medications was unrelated to treatment outcome (Connolly Gibbons et al. 2016).

The parent study sample included 237 patients who were randomized to 16 sessions of either DT or CT. Patients completed 16 sessions in their assigned treatment type. The 16 sessions were delivered over 5 months of treatment (allowing for some missed weeks). Participants were assigned using a computer‐generated urn randomization with a 1:1 allocation algorithm designed by the study statistician. Study conditions were conveyed to the study research assistant at baseline. The study statistician had no contact with the clinical site, and no analyses of the outcome data were conducted until the database was locked. Patients were assigned to therapists with open caseloads.

The present study examines potential severity moderators of treatment effectiveness, with baseline severity examined across five domains, including depressive symptoms, interpersonal problems, mental functioning, physical functioning and quality of life.

#### 1.2.2 Therapists

Therapists employed at the CMHC with a master's degree or above were recruited to treat patients in either dynamic psychotherapy or CT through advertisement at the site. Nine therapists, all female, delivered CT, and 11 therapists, 10 female, delivered STDP. The average length of time therapists had practiced post‐degree was 6.5 years.

### 1.3 Interventions

#### 1.3.1 Cognitive Therapy

The CT implemented in the trial was based on standard CT manuals, consisting of structured sessions focused on both behavioural activation and the exploration of negative thought patterns (Beck 1995; Beck et al. 1979). Specific interventions included evaluating automatic thoughts, behavioural experiments, activity scheduling and subsequent exploration of underlying beliefs and attitudes.

#### 1.3.2 Short‐Term Dynamic Therapy

The STDP implemented in the trial was a supportive‐expressive dynamic psychotherapy (Book 1998; Luborsky 1984). It included both supportive techniques to build a positive working alliance and expressive techniques to help patients gain self‐understanding of their repetitive maladaptive interpersonal patterns. The treatment is an active focused exploration of current relationship conflicts, including explicit socialization to treatment and a focus on specific interpersonal goals.

#### 1.3.3 Training and Supervision

Expert supervisors with extensive experience in delivering and supervising CT or STDP in clinical and research settings provided training and supervision in the current study. All therapists participated in an initial 8‐h training workshop with either the expert CT or STDP supervisor. Therapists then received intensive individual supervision for their first three training cases and attended bimonthly group supervision throughout the study. After a supervisor certified therapists as competent in delivering CT or STDP to two different training cases across at least eight sessions, they were allowed to participate in the randomized trial. Therapists received additional training cases to achieve adequate competence if deemed necessary. Further details of therapist training are provided in Connolly Gibbons et al. (2014) and Connolly Gibbons et al. (2016).

### 1.4 Clinical Evaluators

Nine clinical psychology graduate students provided clinical evaluation while blinded to the treatment condition and study hypotheses. The clinical evaluators administered the Structured Clinical Interview for the DSM‐IV (SCID; First et al. 1997) and the Hamilton Rating Scale for Depression (HAM‐D; Hamilton 1960).

### 1.5 Measures

#### 1.5.1 The Hamilton Depression Inventory (HAM‐D; Hamilton 1960)

The HAM‐D is a widely used inventory for measuring depressive symptom severity. The 17‐item version is an adapted version of the original inventory that incorporates the Structural Interview Guide to enhance reliability (Williams 1988). In the present study, clinical evaluators conducted the HAM‐D at baseline and Months 1, 2, 4 and 5. A meta‐analysis of the HAM‐D reports good interrater and test–retest reliability as well as Cronbach's alpha of 0.79 (Trajković et al. 2011). In the current study, the internal consistencies for the HAM‐D at each of the time points were 0.69 (Baseline), 0.71 (Month 1), 0.75 (Month 2), 0.80 (Month 4) and 0.78 (Month 5).

#### 1.5.2 Inventory of Interpersonal Problems (IIP‐32; Horowitz et al. 2000)

The IIP assesses distressing interpersonal behaviours and difficulties organized as a circumplex along dominance and affiliation axes (Horowitz et al. 2000). We used the 32‐item short form (IIP‐32), which has strong internal consistency (*α* typically 0.80–0.90), test–retest reliability and sensitivity to therapeutic change (Barkham, Hardy, and Startup 1996). Higher scores indicate greater interpersonal distress. The IIP has shown associations with symptom severity, personality pathology and functional impairment and can predict treatment process and outcome (Renner et al. 2012). The IIP was administered at baseline and Months 1, 2, 4 and 5.

#### 1.5.3 Medical Outcomes Study Short‐Form‐36 (SF‐36; Ware and Sherbourne 1992)

The SF‐36 assesses health‐related functioning across eight domains, from which the norm‐based Mental Component Summary (MCS) score and Physical Component Summary (PCS) score are derived (Ware and Sherbourne 1992; Ware et al. 1994). Scores have a mean of 50 (SD = 10) in US norms, with higher values indicating better health. The MCS and PCS show excellent internal consistency (*α* typically 0.87–0.88), test–retest reliability and construct validity across medical and psychiatric populations and are sensitive to clinical change (McHorney et al. 1993; Stewart et al. 1988). Scoring for the MCS and PCS used the orthogonal scoring method based on factor weights. The SF‐36 was administered at baseline and Months 1, 2, 4 and 5.

#### 1.5.4 Quality of Life Inventory (QOLI; Frisch et al. 1992)

The QOLI is a 16‐item self‐report measure that captures overall life satisfaction by having the participant rate the importance of various items in their life such as work, love, friendship, money, health and self‐esteem. Respondents rate the importance of each item on a 3‐point scale and then rate how satisfied they are with the item on a scale that ranges from −3 to 3. By averaging the product of the importance and satisfaction ratings of all items rated as important or extremely important, a composite score for each participant is obtained. Short‐term (2–3 week) test–retest reliabilities of 0.91 (clinical sample) and 0.80 (undergraduate sample) have been reported for the QOLI (Frisch et al. 1992). The QOLI was administered at baseline and Months 1, 2, 4 and 5.

### 1.6 Defining Severity

To illuminate any trends among more severe patients, we defined ‘severe’ using criteria derived from the scientific literature. On the HAM‐D‐17, a score of 24 or above is recognized as severe depression (Zimmerman et al. 2013). For context, Ma et al. (2021) report a mean HAM‐D‐17 score of 18.2 (SD = 7.7) among patients with MDD. This suggests that approximately 16% of those with MDD would have a score of 25.9 or above or a slightly higher percentage at 24.0 or above. Accordingly, we used 24 as our severity cutoff on the HAM‐D‐17. Regarding the IIP‐32, Barkham, Hardy, and Startup (1996) found a mean of 1.5 (SD = 5.1) for a sample of patients presenting for psychotherapy and a mean of 0.98 (SD = 0.52) for a non‐clinical sample. This would suggest that about 16% (1 SD) of those seeking psychotherapy, and about 2.5% (2 SD) of non‐clinical individuals, score above 2.0 on the IIP‐32. We therefore used 2.0 and above as the severity cutoff for the IIP‐32. For the SF‐36 component summary scores, we used a similar 1 standard deviation below the mean of a clinical sample to define severity. Ware et al. (1994) provide mean scores on the SF‐36 MCS (*M* = 37.62) and SF‐36 PCS (*M* = 47.95) on a psychiatric sample for the MOS SF‐36. With a standard deviation of 10, we therefore defined severity as an SF‐36 MCS score less than 27.62 and an SF‐36 PCS score less than 37.95. The QOLI manual (Frisch 1994) provides the following cutoffs for total QOLI scores: very low quality of life (−6.0–0.8), low quality of life (0.9–1.5), average quality of life (1.6–3.5) and high quality of life (3.6–6.0). However, the vast majority of our CMHC sample would meet the definition of very low quality of life based on the 0.8 cutoff. Frisch et al. (2005) present norms for a CMHC sample and found that a score of −1.60 or lower represented the 25th percentile. We therefore used a score of −1.60 or lower to define severely impaired quality of life.

### 1.7 Statistical Analysis

The analysis sample consisted of patients with at least one post‐baseline monthly assessment (*N* = 208). We fit a linear mixed‐effects model predicting HAM‐D change from baseline with HAM‐D‐17 change scores at Months 1, 2, 4 and 5 as the dependent variable. Additional fixed effects were treatment (0 = STDP, 1 = CT), log‐transformed time (log [days from baseline + 1]), the five baseline continuous severity variables as main effects, all two‐way interactions and all three‐way interactions. The model was constructed hierarchically, with main effects evaluated first, then the addition of two‐way interactions and finally the addition of three‐way interactions. Models were implemented specifying random intercepts and random slopes for log‐time and estimated via restricted maximum likelihood, using all available observations under a missing‐at‐random assumption. The primary terms of interest were the treatment × log‐time × baseline severity measure three‐way interactions. The significance level was set at *α* = 0.05 (two‐tailed). A preliminary model was also tested, incorporating the therapist as a clustering variable. However, this model failed to converge with the estimated variance component due to the therapist being equal to zero. We therefore proceeded without incorporating the therapist into the model. To further explore the impact of severity, we graphed trends over time in changes in the HAM‐D‐17 among the subgroup of highly severe patients, for each of the five severity variables, separately by treatment group.

## 2 Results

### 2.1 Characteristics of Samples

There were 208 patients of 237 in the original trial who had at least one post‐baseline score. Of those 208, there were 73 (35.1%) that had a baseline HAM‐D‐17 ≥ 24, 75 (31.1%) that had an IIP‐32 score ≥ 2.0 and 70 (36.1%) that had an SF‐36 PCS score ≤ 37.95. On the SF‐36 MCS and QOLI, however, there were 152 (73.1%) that had a MCS score ≤ 27.62 and 106 (51.0%) with a QOLI score ≤ −1.60, indicating that a large percent of this CMHC sample had more severe mental health functioning and poor quality of life compared with most patients in other psychiatric settings.

Demographic characteristics and mean (SD) on the severity measures, broken down by treatment group, are provided in Table 1. In most cases, the treatment groups were relatively balanced on these variables. The majority of patients were women, single, white and unemployed and completed at least high school/GED. Patients were on average approximately 36 years of age and attended approximately 6.5 sessions of psychotherapy.

**TABLE 1:** Characteristics of patients.

Characteristic	CT *n* = 105	STDP *n* = 103
Female, *n* (%)	79 (75.2)	77 (74.8)
Marital status, *n* (%)		
Never married	58 (55.2)	60 (58.3)
Married/cohabitating	28 (23.8)	15 (17.6)
Separated/divorced	22 (21.0)	23 (22.4)
Relationship status, *n* (%)		
Currently in a long‐term relationship	47 (44.8)	41 (39.8)
Not currently in a long‐term relationship	58 (55.2)	62 (60.2)
Hispanic, *n* (%)	6 (5.7)	2 (1.9)
Race, *n* (%)		
Black/African‐American	43 (40.9)	45 (43.7)
White	52 (49.5)	57 (55.3)
Native American/Alaska Native	2 (1.9)	2 (1.9)
Asian	3 (2.9)	0 (0)
Native Hawaiian or Pacific Islander	1 (1.0)	2 (1.9)
Other/unknown	8 (7.7)	1 (1.0)
Employment, *n* (%)		
Full‐time	6 (5.7)	5 (4.9)
Part‐time	14 (10.5)	5 (4.9)
Stay‐at‐home parent	8 (7.6)	7 (6.8)
Unemployed	54 (51.4)	57 (55.3)
Student	11 (10.5)	6 (5.8)
Disability	15 (14.3)	23 (22.3)
Highest level of education, *n* (%)		
< High school diploma	20 (19.1)	29 (28.1)
High school diploma/GED	33 (31.4)	39 (37.9)
Some college	35 (33.3)	21 (20.4)
College graduate	13 (12.4)	11 (10.7)
Post‐graduate or professional degree	4 (3.8)	3 (2.9)
Age, mean (SD), year	37.1 (12.4)	36.2 (11.8)
HAM‐D‐17 total, mean (SD)	20.9 (5.7)	21.1 (6.0)
IIP‐32 total, mean (SD)	1.7 (0.6)	1.8 (0.6)
SF‐36 MCS, mean (SD)	22.8 (9.2)	22.4 (9.0)
SF‐36, PCS, mean, (SD)	45.3 (12.5)	44.6 (12.1)
QOLI total, mean (SD)	−1.5 (1.9)	−1.4 (2.0)
Number of treatment sessions, mean (SD)	6.3 (5.2)	7.5 (5.3)

The HAM‐D‐17 at baseline was relatively uncorrelated with the other severity measures (Table 2). The IIP‐32 was moderately highly correlated with the SF‐36 MCS (*r* = −0.47) but not the SF‐36 PCS (*r* = 0.07). The SF‐36 MCS and PCS were moderately negatively associated (*r* = −0.38). The QOLI was moderately correlated (*r* = 0.40) with the SF‐36 MCS. These moderate to low correlations reinforce the importance of separately examining severity effects on these measures.

**TABLE 2:** Correlations among severity measures (*N*'s = 202–208).

Subgroup	HAM‐D‐17	SF‐36 PCS	SF‐36 MCS	QOLI
HAM‐D‐17				
SF‐36 PCS	−0.19**			
SF‐36 MCS	−0.09	−0.38**		
QOLI	−0.23**	0.09	0.40**	
IIP‐32	0.21**	0.07	−0.47**	−0.33**

** *p* < 0.01.

### 2.2 Severity Moderators of CT and STDP Slopes

Table 3 provides significance testing for all of the terms in the mixed effects model in a full model. None of the three‐way interactions (severity measured by treatment by log‐time) were statistically significant. An interaction with log‐time was evident for the SF‐36 PCS and was a function of patients with relatively poorer physical health functioning improving at a slower rate than those with relatively better physical health functioning (regardless of treatment type).

**TABLE 3:** Mixed‐effects model significance testing.

Term	F	DF	*p*
HAM‐D‐17	120.59	1,189	< 0.001
Treatment group (Rx)	0.69	1,183	0.406
Logdays	21.43	1,131	< 0.001
Rx * logdays	0.15	1,127	0.701
Logdays * HAM‐D‐17	0.34	1,136	0.568
Rx * HAM‐D‐17	0.06	1,131	0.805
Rx * logdays * HAM‐D‐17	0.194	1,132	0.661
IIP‐32 total	1.11	1,170	0.293
Logdays * IIP‐32 total	2.74	1,122	0.101
Rx * IIP‐32 total	1.29	1,167	0.127
Rx * logdays * IIP‐32 total	0.039	1,121	0.844
QOLI total	0.19	1,190	0.667
Logdays * QOLI total	2.76	1,131	0.099
Rx * QOLI total	0.79	1,185	0.374
Rx * logdays * QOLI total	0.115	1,129	0.735
SF‐36 MCS	0.18	1,200	0.674
Logdays * SF‐36 MCS	1.08	1.147	0.301
Rx * SF‐36 MCS	1.32	1,195	0.252
Rx * logdays * SF‐36 MCS	0.120	1,145	0.729
SF‐36 PCS	15.53	1,185	< 0.001
Logdays * SF‐36 PCS	5.79	1,126	0.018
Rx * SF‐36 PCS	0.15	1,180	0.703
Rx * logdays * SF‐36 PCS	0.054	1,125	0.816

**FIGURE 1:**
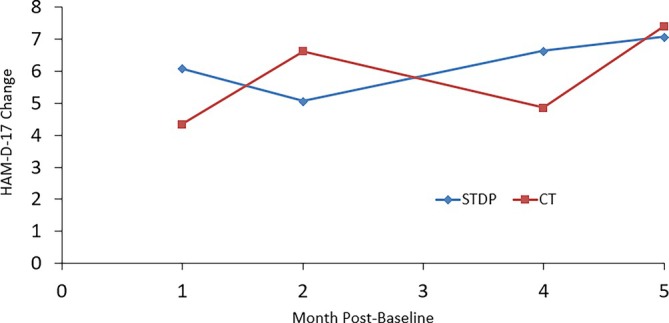
Model‐based estimated change in HAM‐D‐17 total scores over time for patients ≥ 24 on baseline HAM‐D‐17.

**FIGURE 2:**
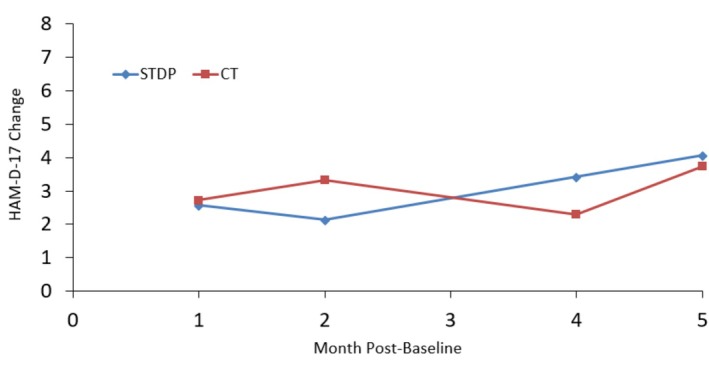
Model‐based estimated change in HAM‐D‐17 total scores over time for patients ≥ 2 on baseline IIP‐32 total score.

**FIGURE 3:**
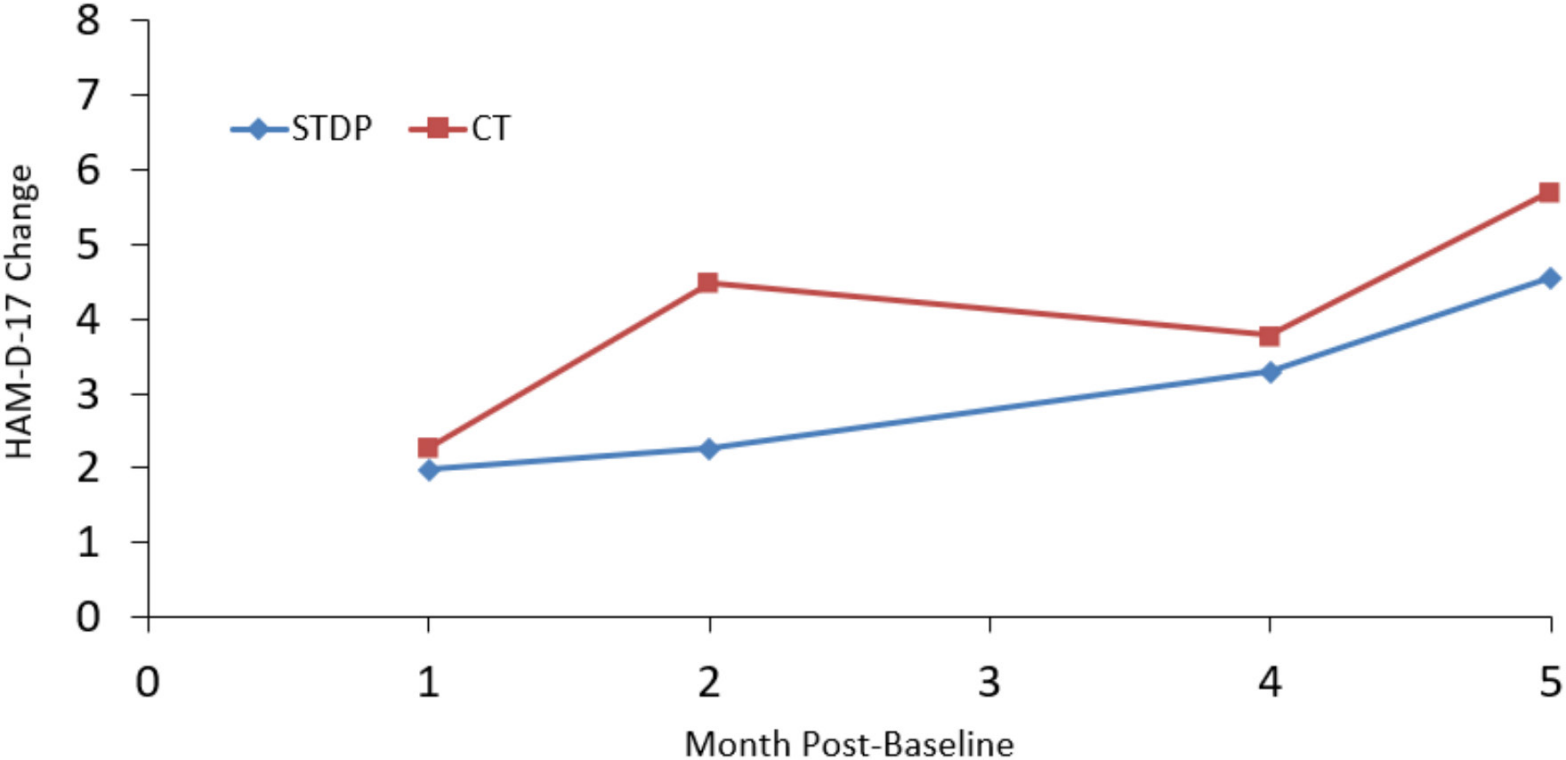
Model‐based estimated change in HAM‐D‐17 total scores over time for patients ≤ 27.6 on baseline SF‐36 mental component summary score.

**FIGURE 4:**
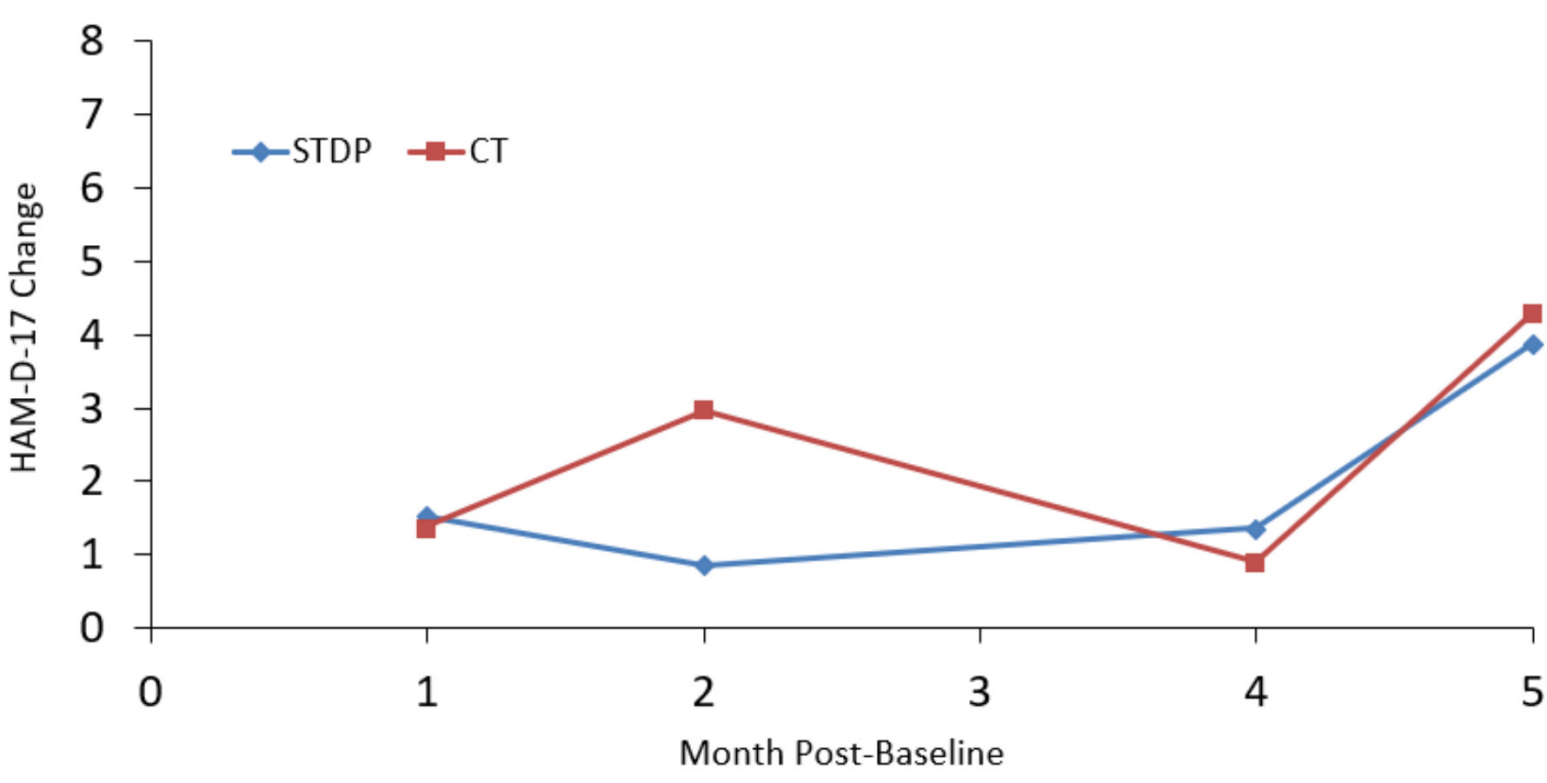
Model‐based estimated change in HAM‐D‐17 total scores over time for patients ≤ 37.4 on baseline SF‐36 physical component summary score.

**FIGURE 5:**
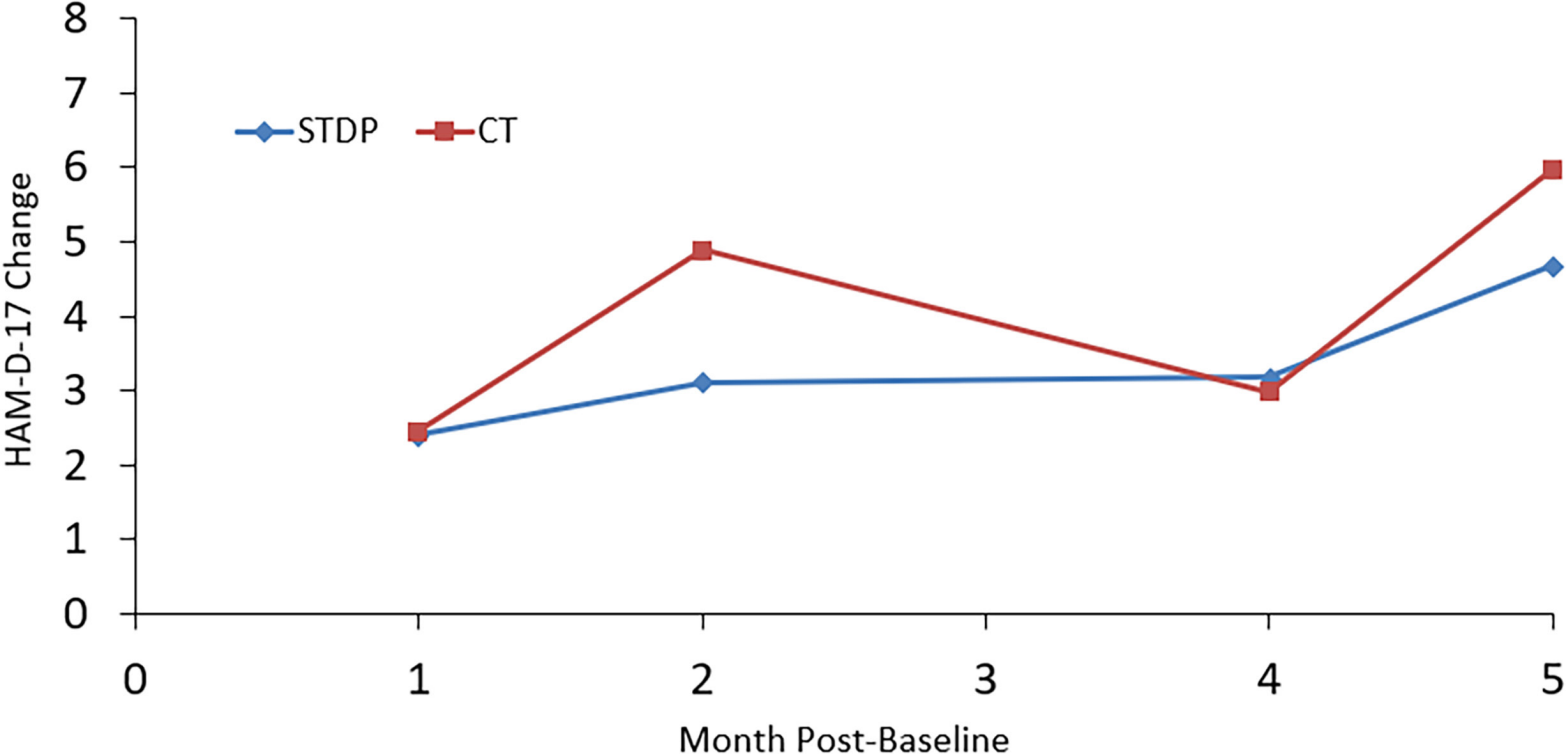
Model‐based estimated change in HAM‐D‐17 total scores over time for patients ≤ −1.60 on baseline QOLI total score.

Figures 1, 2, 3, 4 and 5 show observed (raw) HAM‐D‐17 mean changes at the monthly assessments within each high severity subgroup, with separate lines for CT and STDP. Effect sizes calculated on the last observed HAM‐D‐17 change for each severity definition were HAM‐D‐17 (*d* = −0.03), IIP‐32 (*d* = −0.13), SF‐36 MCS (*d* = 0.16), SF‐36 PCS (*d* = −0.06) and QOLI (*d* = 0.23). All effect sizes were small or trivial. The direction of effects however was in line with hypotheses for the SF‐36 MCS and QOLI (CT greater improvement than STDP) and the IIP‐32 (STDP greater improvement than CT).

## 3 Discussion

This secondary analysis of a clinical trial database evaluated whether five dimensions of severity moderated the effects of CT compared with STDP. By incorporating interpersonal problems, functioning and health‐related quality of life indicators, these results extend symptom‐based approaches to evaluating severity.

For the full sample, there were no significant three‐way interactions (severity variable by treatment by log‐time). Thus, there was no evidence of moderation of treatment effectiveness across the range of severity on each severity measure. Among the most severe, subgroup comparisons of CT and STDP yielded effect sizes that were small or trivial. These results, therefore, provide no evidence for the hypothesized superiority of CT over STDP for individuals with severe depressive symptoms or for individuals with severe impairments in mental or physical functioning or quality of life. The results also fail to confirm the hypothesis that STDP is superior to CT for individuals with severe interpersonal problems. As such, our findings are in general consistent with the meta‐analysis of Gómez Penedo, Meglio, et al. (2025) that failed to find differences between psychotherapies for depression in the association between baseline severity of interpersonal problems and change in depressive symptoms. The current findings extend Gómez Penedo, Meglio, et al.'s (2025) results by specifically examining CT and STDP.

It is important to note that the degree of absolute HAM‐D‐17 change was modest among the most severe. Starting treatment with a mean baseline HAM‐D‐17 score of about 27, a raw mean change of about 7 points was evident at Month 5 for both CT and STDP within the baseline HAM‐D‐17 ≥ 24 subgroup. This results in a Month 5 HAM‐D‐17 score of about 20, still in the range of moderate severity. This is in comparison with efficacy trial results for CT showing a HAM‐D‐17 change of approximately 8 points from a baseline score of 23 to an end score of 15 over the course of 8 weeks in a moderate to severe depression sample (DeRubeis et al. 2005). Thus, our 7‐point change for CT is not that discrepant from the 8‐point change extrapolated from Figure 1 in DeRubeis et al. (2005). Though the current study had a full treatment period of 16 weeks of treatment, the number of treatment sessions attended was 6.5 on average. In the DeRubeis et al. (2005) trial, we estimate an average number of treatment sessions of about 7 (this is based on the reported 15% attrition), and therefore, the DeRubeis et al. (2005) trial had slightly more change in CT but with a slightly longer average duration of treatment. Further, the DeRubeis et al. (2005) efficacy trial had more restrictive inclusion/exclusion criteria and employed doctoral‐level, highly trained CT therapists. Moreover, the Connolly Gibbons et al. (2016) sample had a patient population with high levels of trauma and medical/psychiatric comorbidity that may have limited the absolute amount of change (Goldstein et al. 2023). The impact of these factors is evident in the noticeably smaller change on the HAM‐D‐17 within the high severity groups defined by interpersonal severity as well as physical functioning severity (HAM‐D‐17 change of about 4 points by Month 5). All of these factors may have contributed to the slightly lower amount of raw change on the HAM‐D‐17 in the high severity subgroups found here compared with efficacy study results within the HAM‐D‐17 severity subgroup. Despite the modest amount of change for CT on the HAM‐D‐17 in the DeRubeis et al. (2005) study, an important finding of that study was that CT was as effective as medication in the acute treatment of moderate to severe depression. Whether STDP would be noninferior to medication in the treatment of severe depression remains to be evaluated in a prospective clinical trial.

By including multiple clinically important definitions of severity, our study extends previous MDD studies that have a more limited focus only on depressive symptom severity. It has been argued that overall quality of life and functioning is of central clinical significance, especially to patients, given that a variety of studies have suggested that patients' subjective well‐being is often more relevant than their objective medical condition in determining their compliance and evaluation of treatment (Gladis et al. 1999; Hunt and McKenna 1993). Many patients seek psychotherapy treatment specifically because of family or social problems (Nakash et al. 2018). Though a focus on psychiatric disorders will likely remain the central priority for clinical trials, adding an additional clinical trial focus on the issues important to patients, such as severe impairment in quality of life and/or severe interpersonal problems, may yield new ways of providing optimal outcomes that increase patient satisfaction with treatment. Given the noticeably smaller improvement in depressive symptoms evident for both treatment modalities within the severe interpersonal problems and physical functioning subgroups, more attention is needed to develop and investigate alternative models of treatment for such patients. Future study designs may include longer treatment durations or additional elements to treatment models to address severe interpersonal problems and poor physical functioning.

In terms of future studies, there may be additional severity variables not examined here that could be particularly relevant for understanding differential treatment effects. For example, the particular type of interpersonal problems at baseline has been shown to be related to differential treatment outcomes. In one study of chronic depression, greater severity on the IIP vindictive/self‐centred subscale was associated with better outcomes in mindfulness‐based cognitive therapy (MBCT) than in the cognitive‐behavioural analysis system of psychotherapy (CBASP), whereas greater severity on the IIP non‐assertive subscale was associated with a better outcome in CBASP than in MBCT (Probst et al. 2020).

An important limitation of the current study is that it was a post hoc analysis. In addition, the lack of a control condition prevents ruling out that the effects for either treatment were a result of the passage of time. Another important limitation is that these results may generalize only to the community mental health setting. Our post hoc subgroup analysis of the Connolly Gibbons et al. (2016) data does not inform whether these treatments would have comparable outcomes in settings using more expert therapists or with different patient populations. Given the selection of a subgroup of patients from the randomized sample, it is possible that unmeasured confounding may have biased treatment comparisons. Such confounding can occur even with randomization, though the parent study used an urn randomization method with seven factors and found the treatment groups to be balanced on observed pre‐treatment variables.

In conclusion, this post hoc analysis found no evidence of severity moderators of outcome differences between CT and STDP among patients, using five domains of severity: depressive symptoms, interpersonal problems, mental functioning, physical functioning and quality of life. Future prospective studies with greater statistical power are needed to confirm these results and extend treatment comparisons beyond a focus on symptoms.

## Ethics Statement

The original trial was approved by the University of Pennsylvania's Institutional Review Board committee #8. Informed consent was obtained from all participants in the study.

## Conflicts of Interest

The authors declare no conflicts of interest.

## Data Availability

The code and data can be made available upon reasonable request to the corresponding author, Dr. Mary Beth Connolly Gibbons (gibbonsm@pennmedicine.upenn.edu).
